# European Registry of Materials: global, unique identifiers for (undisclosed) nanomaterials

**DOI:** 10.1186/s13321-022-00614-7

**Published:** 2022-08-24

**Authors:** Jeaphianne van Rijn, Antreas Afantitis, Mustafa Culha, Maria Dusinska, Thomas E. Exner, Nina Jeliazkova, Eleonora Marta Longhin, Iseult Lynch, Georgia Melagraki, Penny Nymark, Anastasios G. Papadiamantis, David A. Winkler, Hulya Yilmaz, Egon Willighagen

**Affiliations:** 1 Department of Bioinformatics—BiGCaT, NUTRIM, FHML, Maastricht University, Maastricht, The Netherlands; 2 NovaMechanics Ltd., 1070 Nicosia, Cyprus; 3 Sabanci University Nanotechnology Research and Application Center (SUNUM), Tuzla, 34956 Istanbul Turkey; 4 Health Effects Laboratory, Department of Environmental Chemistry, Norwegian Institute for Air Research, 2007 Kjeller, Norway; 5 Seven Past Nine d.o.o., 1380 Cerknica, Slovenia; 6 Ideaconsult Ltd., Sofia, 1000 Bulgaria; 7 School of Geography, Earth and Environmental Sciences, University of Birmingham, Edgbaston, B15 2TT UK; 8 Institute of Environmental Medicine, Karolinska Institute, Stockholm, Sweden; 9 School of Biochemistry and Chemistry, La Trobe Institute for Molecular Science, La Trobe University, Bundoora, Australia; 10 Monash Institute of Pharmaceutical Sciences, Monash University, Parkville, Australia; 11 School of Pharmacy, University of Nottingham, Nottingham, UK

**Keywords:** Nanomaterial, FAIR, Identifier

## Abstract

Management of nanomaterials and nanosafety data needs to operate under the FAIR (findability, accessibility, interoperability, and reusability) principles and this requires a unique, global identifier for each nanomaterial. Existing identifiers may not always be applicable or sufficient to definitively identify the specific nanomaterial used in a particular study, resulting in the use of textual descriptions in research project communications and reporting. To ensure that internal project documentation can later be linked to publicly released data and knowledge for the specific nanomaterials, or even to specific batches and variants of nanomaterials utilised in that project, a new identifier is proposed: the European Registry of Materials Identifier. We here describe the background to this new identifier, including FAIR interoperability as defined by FAIRSharing, identifiers.org, Bioregistry, and the CHEMINF ontology, and show how it complements other identifiers such as CAS numbers and the ongoing efforts to extend the InChI identifier to cover nanomaterials. We provide examples of its use in various H2020-funded nanosafety projects.

**Graphical Abstract:**

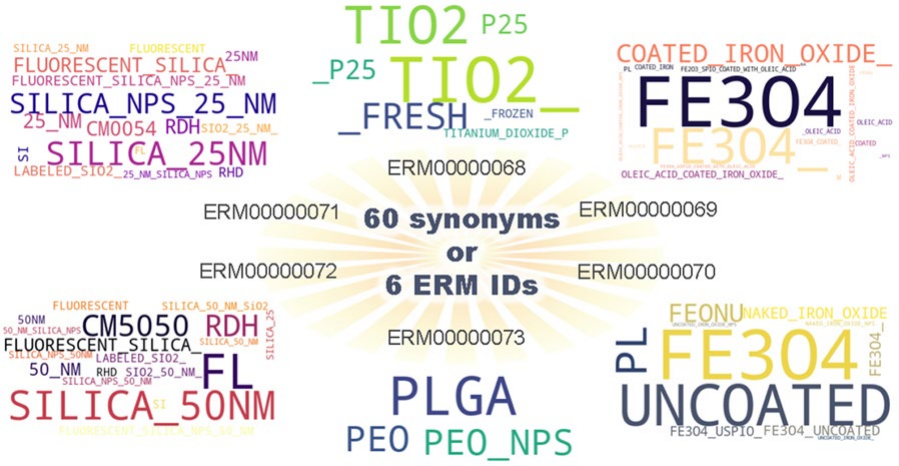

## Introduction

Management of nanomaterial and nanosafety data is essential for development of a transparent risk governance model in which decisions are based on high quality datasets that can be appropriately cited [1, 2]. This requires a significant initial effort but, in the medium term, the enhanced re-usability of nanosafety data will save the European community a significant amount of money [3]. Recently, the FAIR approach for data findability, accessibility, interoperability, and reusability was formulated to optimize the utility of decades of research in these areas [4, 5]. A central element of the FAIR resource output is the global, unique identifier, the workhorse of data integration for many years.

Examples of such identifiers for chemicals include the Chemical Abstracts Service (CAS) registry number [6] and the International Union of Pure and Applied Chemistry (IUPAC) International Chemical Identifier or InChI [7]. The first is assigned to all chemical substances after publication and indexing, while the second can be computed according to an agreed set of rules and abstractions to represent chemical structures. However, these two identifiers are still not sufficient for the wide range of nanosafety research projects being undertaken. The InChI is based on chemical graphs and cannot fully describe the chemistry of engineered nanomaterials (ENMs), although a draft extension of InChI for nanomaterials (NInChI) has been defined recently [8]. The goal of the NInChI is to describe core features of nanomaterials (from their center towards their surface, the aspect most closely linked to their interactions and potential toxicity). Its purpose is to support integration of datasets of similar materials and, as such, is considered a group identifier. CAS registry numbers cannot be created by researchers themselves. This is an issue for internal communication and reporting in (European) research projects where the full physicochemical properties are often measured, but not always reported outside the project, with scientific publication often coming well after the project has ended. Thus, to meet the FAIR requirements, we need a complementary global, unique identifier for unpublished or internally published communication and reporting that can be used without disclosing sensitive or embargoed information, but which allows linking of knowledge in a clearly defined way to support nanosafety research and development [9].

Here we describe a recent initiative of several EU NanoSafety Cluster projects to establish such an identifier for use within projects and beyond, modelled on similar efforts in the pharmaceutical industry. In the case of pharmaceuticals, chemicals reported by a company receive an identifier without the need to disclose their structures [10]. This identifier can be used in, for example, clinical trials and publications, thus linking data to the unique registry number for the specific chemical as information becomes public [11]. Applying this approach to nanomaterials and nanosafety research, projects would compile the list of nanomaterials that they will use and “register” these on behalf of the project. Each nanomaterial then receives an identifier, which may be accompanied by additional information such as supplier, composition, size and batch number, or can simply be a placeholder that does not disclose their structure or composition. This identifier can be used in project (deliverable) reports and subsequent publications, for example. At some point the chemical structure is disclosed and the identifier is linked to the identity of the material. While this expectation is not explicitly required, releasing data is the default for European Horizon 2020 projects.

We here report the development of a public registry where nanomaterial identifiers can be registered, the European Registry of Materials (nanocommons.github.io/identifiers/). The project is currently governed by the European Horizon 2020 NanoCommons project and the registry itself is available under a CCZero license, removing obstacles for reuse.

## Materials and methods

The registry contains supporting pages written in Markdown, [12] hosted on GitHub (github.com/nanocommons/identifiers), and a central registry of materials in Turtle [13] (github.com/NanoCommons/identifiers/blob/master/registry). The webpage was autogenerated by GitHub Pages. The European Registry of Materials Identifier was registered with FAIRSharing [14] and identifiers.org [15]. The latter provided us with an official compact identifier structure [16]. The registry Turtle uses the RDF Schema specification for storing labels and types of nanomaterials as *chemical substances*, using the CHEBI_59999 term from the ChEBI ontology [17]. The registry is released to Zenodo several times a year to make the repository more FAIR. This release process is supported by the CITATION.cff in the GitHub repository.

## Results

### The European Registry of Materials identifier

We established the European Registry of Materials (nanocommons.github.io/identifiers/) in April 2019, allowing us to create global, unique identifiers to track nanomaterials used in individual research projects with minimal required information upfront. This provides users with full control over when to disclose or otherwise provide additional information on specific nanomaterials and accompanying physico-chemical and toxicological data. It allows users to discuss nanomaterials in a FAIR way, even before the detailed chemistry of the nanomaterials has been established. At the time of writing, six nanosafety projects have registered materials with the registry: NanoSolveIT, NanoFASE, RiskGONE, NanoTest, caLIBRAte, and SbD4Nano. Additionally, the European Gov4Nano project implemented these identifiers in several data reuse case studies, though no materials have been registered at the time of writing this paper.

The current number of European Registry of Materials Identifiers (or ERM identifiers for short) for registered materials is > 450. The European Registry of Materials identifier is registered with FAIRSharing at fairsharing.org/bsg-s001384/. The registration of the identifier in identifiers.org is available at registry.identifiers.org/registry/erm (MIR:00000763) and in Bioregistry at bioregistry.io/registry/erm. This also defined the ‘erm’ namespace for the compact identifier. Finally, the ERM identifier has also been added to the Chemical Information Ontology (CHEMINF) [18] and is included in the eNanoMapper ontology [19], allowing encoding of the ERM identifiers in Resource Description Framework representations of nanosafety data and annotation of ERM identifiers in spreadsheets and ISA-Tab files [20].

For example, the NanoSolveIT project registered a material with the ERM00000001 identifier. The full Uniform Resource Identifier (URI) for this compound is https://nanocommons.github.io/identifiers/registry#ERM00000001 which is too long to be used in documentation. The corresponding compact identifier erm:ERM00000001 is easy to use in written material, analogous to the use of Protein Data Bank (PDB) identifiers for proteins in journals.

### Registering identifiers

Registration of new identifiers ultimately occurs by adding the new identifier and a label from the project assigning the identifier. The label can be a material name, but also something generic such as “nanomaterial 1”. The label and information about the project that created the ERM identifier are the minimal mandatory pieces of information about a registered material. Eventually a lot more information should become available for each registered identifier. One way to do this is to add a link to a (GitHub) page with more information. An example of a registry entry for one of the identifiers in Turtle format containing such a link is (the namespaces are defined as abbreviations at the end of the manuscript):
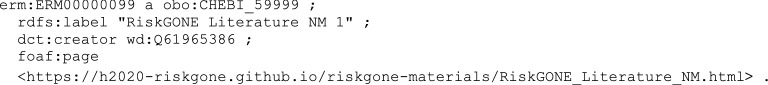


Other information that can be provided includes a parent ERM identifier, the chemical composition, a batch number, an ontology material classification (using the eNanoMapper ontology), a web page, and a provider, contact, or project name. A parent ERM identifier can be useful when a material is a modification of the parent, or when a material is from a specific batch while the parent is anything that has been synthesised in the same way. If an ontology classification does not currently exist for a specific nanomaterial, users can request it through the eNanoMapper ontology GitHub page github.com/enanomapper/ontologies/issues/new/choose [19].

ERM identifiers for materials can be registered in two ways:

(i) By creating a request in the GitHub project issue tracker, as explained in a dedicated tutorial at nanocommons.github.io/identifiers/register; or
(ii) By communication (e.g. email or pull requests) to the NanoCommons team, which is the way most current identifiers have been registered. The second approach works best when multiple identifiers need to be registered together, e.g. at the initial stages of a new project when decisions around the nanomaterials to be utilised in the project are finalised.

### The ERM and FAIR

The ERM helps projects consider the FAIR principles from the start. The ERM identifiers are part of the metadata and can be used to indicate that different documents and datasets are talking about the same material. Knowledge and data about the material behind a specific ERM is not defined in nor provided by the registry, but comes from the project registering the identifier and from experimental and computational data later published about the material referencing the ERM identifier. A unique global identifier is essential to link all information and other metadata, ensuring FAIR dissemination of the knowledge about that material. Having the ERM identifier before the (meta)data is shared and the first experiment is commenced helps the projects manage their internal data, with the long term goal of making data availability open and FAIR.

As well as helping others make their data FAIR, the ERM identifier itself also needs to be FAIR. Table 1 below shows how the ERM is currently addressing all the FAIR principles, both intrinsically and as a FAIR solution to others. The current levels of FAIR of the ERM identifier and the ERM registry will continue to be improved, e.g. by collaborating with GO FAIR networks and defining a FAIR Implementation Profile (FIP).

**Table 1:** Description for each maturity indicator of how the ERM is FAIR and how it can be used to make nanosafety data FAIR

	FAIR Maturity Indicators	FAIRness of the ERM identifier and registry	How the ERM can make data FAIR
Findability	F1. (Meta)data are assigned a globally unique and persistent identifier	The registry is released to Zenodo, which provides it with a DOI	The ERM identifier is a globally unique and persistent identifier; The registry supports depositing metadata
	F2. Data are described with rich metadata (defined by R1 below)	The ERM identifier is registered with FAIRSharing at fairsharing.org/bsg-s001384/, in identifiers.org at registry.identifiers.org/registry/erm (MIR:00,000,763), and in Bioregistry at bioregistry.io/registry/erm	The ERM is one important aspect of rich metadata and helps make metadata findable across resources
	F3. Metadata clearly and explicitly include the identifier of the data they describe	The registry archive uses the DOI to point to that version of the registry content	The ERM identifier can easily be included in spreadsheets and as Compact Identifiers in reports
	F4. (Meta)data are registered or indexed in a searchable resource	Zenodo records and DOIs are indexed by many services	Some metadata can be shared in the registry, but with the ERM identifier further metadata can also be provided via other channels
Accessibility	A1. (Meta)data are retrievable by their identifier using a standardised communications protocol	GitHub and Zenodo use HTTP	
	A2. Metadata are accessible, even when the data are no longer available	Zenodo guarantees availability for 20 years	
Interoperability	I1. (Meta)data use a formal, accessible, shared, and broadly applicable language for knowledge representation	The registry uses the RDF specification and all instances are annotated with ontologies	ERM identifiers are URIs and can be expressed as Compact Identifiers
	I2. (Meta)data use vocabularies that follow FAIR principles		The ERM is FAIR and by using it, users implement I2
	I3. (Meta)data include qualified references to other (meta)data	The registry explicitly encourages users to link to other resources, including webpages and projects	The global unique ERM identifier enables linking (meta)data
Reusability	R1. Meta(data) are richly described with a plurality of accurate and relevant attributes	The registry is available under a CCZero license. The ERM identifiers specification is documented on GitHub and archived on Zenodo	By adding the global unique ERM identifier to research output, reuse is facilitated. The ERM identifiers simplify tracking the provenance of nanomaterials and their properties

### Use of ERM identifiers by EU NanoSafety Cluster projects

The EU Nanosafety Cluster (NSC, www.nanosafetycluster.eu/) is a self-organising group of projects addressing various aspects of nanomaterials safety that has been operating since the start of the 7th Framework Programme [21]. Its exact composition varies as projects finish and new ones begin, but its collaborative activities are maintained through a set of overarching working groups, including WG-F on data management, which supports the projects in aspects of FAIR data and promotes the ERM. This section describes some of the current NSC projects (in no particular order) that have registered ERM identifiers, how they are using them, and projects that are actively involved with supporting community adoption of the identifier.

#### NanoCommons

NanoCommons (www.nanocommons.eu/) is the nanosafety infrastructure project whose goal is to build a common data management environment across European and international nanosafety projects. It does not generate its own data so ERM identifiers have not been registered. However, as a cross-project enabler it accepted the responsibility to develop the ERM and host it on the NanoCommons GitHub. Additional community support activities undertaken by NanoCommons include extending the common ontology, the eNanoMapper ontology, which has been maintained by NanoCommons in recent years [22]. The ERM identifier aligns well with this goal as it allows nanosafety research projects to globally and uniquely refer to specific nanomaterials. NanoCommons is also driving the development of the NInChI which aims to provide the means by which specific information related to individual nanomaterials in the ERM can be added, and similarity of materials in the registry can be assessed.

**Fig. 1:**
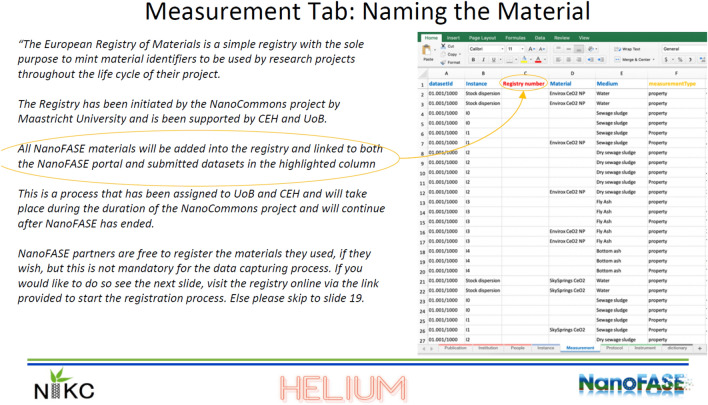
Implementation of the ERM identifiers in the revised NIKC templates used in the NanoFASE project and inclusion in the guidelines document

Furthermore, via its Transnational Access activities (www.nanocommons.eu/ta-access/) NanoCommons was responsible for guiding the data management processes of the NanoFASE project. In this way, we were able to promote the use of ERMs for the nanomaterials used within the project. The use of ERMs as a unique identifier for the project’s nanomaterials was included, as a provision, in the revision and updating of the adopted Nanoinformatics Knowledge Commons (NIKC, www.ceint.duke.edu/research/nikc, [23]) data curation templates (see Fig. 1) and relevant guidance was included in the guides developed through the collaboration of the NanoFASE and NanoCommons projects [24], including the international partners Center for the Environmental Implications of Nanomaterials (CEINT) of Duke University and Team Helium LLC (U.S.A.).

**Fig. 2:**
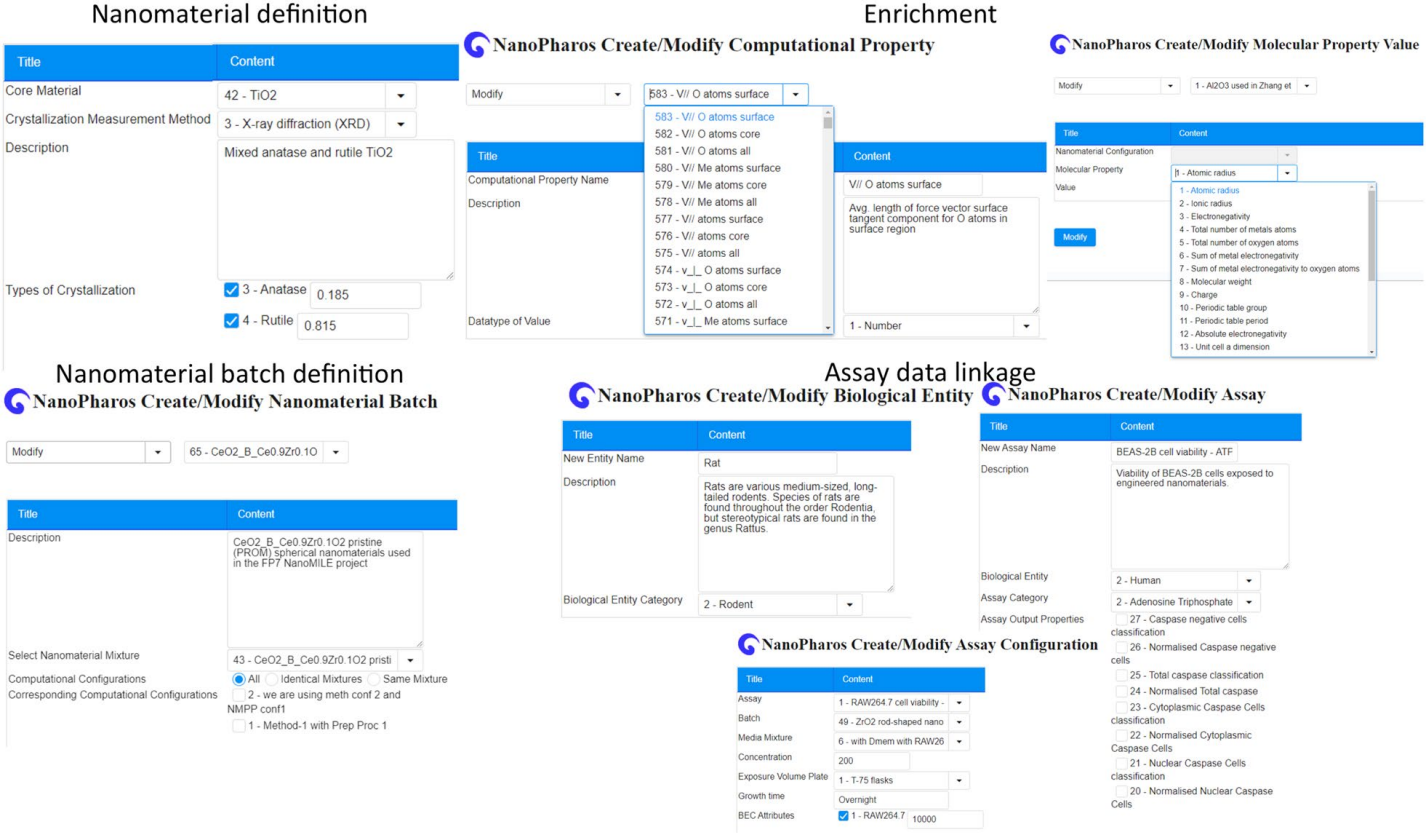
The nanoPharos database offers users the ability to define specific nanomaterials, enrich the main structure with molecular and atomistic descriptors, which can be linked with a specific ERM. The system offers the ability to define separate batches of the same nanomaterial, directly linked to the original material and to the subsequent biological assays. In this way, it is possible to track the lifecycle of the starting nanomaterial and its different batches using the ERM

#### RiskGONE

RiskGONE (www.riskgone.eu/) has registered seventeen ERM identifiers for the nanomaterials selected for use in the round robin validation exercises for human and environmental health assay pre-validation. Within the project, these identifiers are linked to a label (currently “RiskGONE NM 1–17”), supplier, supplier code, and batch or vial number. This information is kept in a repository (h2020-riskgone.github.io/riskgone-materials/identifiers_and_guidance.html) as a versioned Markdown file, with Word and PDF exports for convenience. The ERM identifiers are used in the project`s documents, deliverables and reports and are integrated in the eNanoMapper database data entry workflow [1, 2]. The RiskGONE data entry templates are following the current Nanosafety Cluster practice of using Excel templates for data logging. Physico-chemical characterization data generated by project partners is entered into a NANoREG style template for physchem properties [25, 26] and data from in vitro toxicity tests for human and eco-toxicological hazard assessment is entered into adapted IOM–Nano–EHS data templates [1]. These templates have been developed over the last decade and used by a number of EU FP7 and H2020 projects. All templates are distributed through an online Template Wizard tool (search.data.enanomapper.net/projects/riskgone/datatemplates/), and include a dedicated “Materials” sheet, which is automatically filled in with the ERM identifiers, names, types, and other details of the materials used in the project. This spreadsheet serves as a lookup for selecting the materials on data entry and ensures consistent use of material identifiers across the project.

An additional 196 ERM identifiers are used in a data extraction project in collaboration with the SbD4Nano project (www.sbd4nano.eu/). Data was extracted for all nanomaterials mentioned in 21 papers that were previously analyzed for adverse outcomes [27]. This resulted in 196 nanomaterials with matching ERM identifiers. The meta-data for these ERM identifiers, made freely available by the RiskGONE project, can be found here: h2020-riskgone.github.io/riskgone-materials/RiskGONE_Literature_NM.html. The nanomaterials and their Molecular Initiating Events (MIEs) are captured in an RDF schema and annotated using standard metadata vocabularies and specialized ontologies (manuscript in preparation). This way, the identifiers are linked to a nanomaterial name, the reference from which the data is extracted, the PhysChem properties of the nanomaterial as reported in the publication and the MIE, Key Event and/or Adverse Outcome Pathway reported for that nanomaterial. This exemplifies how the ERM identifier can be used to discuss nanomaterials in a FAIR way.

#### Gov4Nano

The Gov4Nano project (www.gov4nano.eu/) recently established the GO FAIR implementation network, AdvancedNano (www.go-fair.org/implementation-networks/overview/advancednano/) supports implementation of the FAIR principles in nanosafety data management systems. To achieve the overarching aim to disseminate and collaborate on solutions for increasing FAIRness of nanosafety data, the project is working on a set of six case studies that demonstrate findability and accessibility (case study 1), interoperability (case study 2), and reusability (case studies 3 to 6) of nanosafety data. Case study 1 is focused on implementation of persistent identifiers and aims to develop strategies for the community to actively use such identifiers, including the ERM identifiers, focusing on barriers and incentives for uptake of solutions. Case study 2 focuses on the use of electronic laboratory notebooks to support increased implementation of workflows to standardize data formats and harmonize metadata annotation, including ERM identifiers. This relates to work developed in NanoCommons on the data life cycle and metadata requirements and needs at all stages of the nanosafety data lifecycle [28]. Case studies 3 to 6 aim to demonstrate reuse of omics, ecotoxicological, genotoxicity, and human occupational exposure data, respectively. Each case study will consider curation strategies involving implementation of ERM identifiers. For example, the omics case study is focused on providing reusable nanosafety-relevant omics data as input for tools and models in the Nano Risk Governance Portal. This supports the Nano Risk Governance Council that was developed in concert with the two other governance projects (RiskGONE and NanoRIGO). Reuse of omics data is highly dependent on integration with other types of data, such as physicochemical and exposure data. Implementation of the ERM identifiers supports this type of integration across diverse types of data and eventually enables interoperable reuse of relevant high-content omics data [2].

#### NanoSolveIT

The nanoPharos database (db.nanopharos.eu/), developed within the H2020 NanoSolveIT (www.nanosolveit.eu, [29]) and NanoCommons projects, promotes accessibility, interoperability and reusability of curated datasets in a ready-for-modelling format. The nanoPharos database has been designed under the FAIR data principles to also include computationally derived data from simulations of nanomaterials at different levels of complexity. The database was further extended to include nanomaterials characterization data that can be enriched with a series of atomistic, molecular and structural descriptors. The database offers the possibility of including different batches and instances of the same nanomaterial, so as to monitor any physico-chemical transformations across its entire lifecycle. Thus, it is possible to use a single starting ERM and follow nanomaterial transformations/changes in different laboratories and/or projects and under different environmental conditions. The nanomaterials data can be linked with biological effects data to support complete in silico nanosafety evaluation. Currently, the NanoSolveIT project has registered 57 ERM identifiers to uniquely identify which nanomaterials the information in the database relates to. An additional 24 ERM identifiers (erm:ERM00000277-erm:ERM00000300) were added to the nanoPharos database for a dataset derived from a recent publication [30]. Because this dataset was a subset of the original dataset, which was in NanoWiki 6 [31], the ERM identifiers were also added to NanoWiki. To enable reporting of materials in the NanoSolveIT project, an additional 69 and 63 ERM identifiers were added to project spreadsheets and metadata was captured in private GitHub repositories (Fig. 2).

#### caLIBRAte

A total of nine ERM identifiers were used in the caLIBRAte​ project. The focus of the project was on specific NRCWE nanomaterials that already had their own identifiers. These NRCWE IDs were coupled to ERM identifiers in the caLIBRAte—eNanoMapper database at search.data.enanomapper.net/projects/calibrate. With this mapping (currently internal to the project), the ERM identifiers provide information on the chemistry, state and supplier of the nanomaterials.

#### SbD4Nano

The SbD4Nano project initially involves 10 preliminary engineered nanomaterials (ENMs). As each nanomaterial is surface modified, an ERM identifier number will also be assigned for the new materials. The registered identifiers for the preliminary ENMs are labeled with batch number, date, chemical composition, supplier name, and code using eNanoMapper (search.data.enanomapper.net/projects/sbd4nano/). Within the project, toxicological profiles of ENMs are investigated in vitro. The eNanoMapper database [2] is also used for toxicological data management for the modified ENMs. This information is made available to other project partners through email, databases, and as Markdown files. The properties of ENMs such as substance identification, substance and physicochemical characterization, matrix information, end-product information, sector applications, and process of synthesis from the supply chain participants, were registered on the shared project drive. Data generated by project partners is being entered using NanoSafety cluster Excel templates, with an integrated Materials datasheet, allowing easy ERM lookup during data entry, as described for the RiskGONE project database. While the data templates are shared between projects, the TemplateWizard populates the Materials datasheet with project-specific material identifiers.

For the SbD4Nano project, data extraction will be done in the same way as for the RiskGONE project (see "RiskGONE" section). Six ERM identifiers have already been used for this effort.

#### NanoTest

The FP7 NanoTest project (www.nanotest-fp7.eu) focused on ENMs used in nanomedicine. As the area of nanomedicine brings humans into direct contact with nanomaterials, data on ENM interaction with cells from eight different organs and tissues were studied. The NanoTEST project is complete and data is available to several ongoing projects (e.g. RiskGONE) through the eNanoMapper Nanosafety Data Interface [2] search.data.enanomapper.net/. Six materials are linked to ERM identifiers, ERM00000068-ERM00000073. Genotoxicity, oxidative stress, cell viability, immunotoxicity and physicochemical characterisation data generated on blood, lung, brain, gastrointestinal tract, liver, kidney, vascular system and placenta models by the project partners can be retrieved using these identifiers.

## Discussion

Seven nanosafety projects have begun using the ERM identifier, a great start. Clearly, not all questions on how the identifier will be used in practice have been answered as yet. Indeed, during the initial dissemination of the identifier, a number of questions arose. For example, do we really need another identifier? As indicated in the introduction, the identifiers that are currently available have limitations. The Registry does not bar the creation of identifiers for nanomaterials that already have an identifier, however the purpose is primarily to create identifiers for materials that do not yet have a unique identifier.

A second question arose about the anonymity of the materials. As with the previous question, the Registry allows users to register only a label and creator, however the Registry can host several additional types of optional information. The reason for this choice is that when we start research on a new material, we may not have established its identity, but we need to ensure we order and/or distribute the correct material. The new identifier serves that purpose.

A third issue is that the same materials may be registered more than once. This can happen when projects that operate independently and are not aware of each other, continue to conduct research on materials from a completed project. However, this problem is common to other identifier systems, and the equivalence of identifiers and how to handle it, has been studied [32, 33]. A practical solution to this problem is to simply use an identifier mapping showing that two identifiers refer to exactly the same material. It is important to realize that this approach is a lot more tractable than accidentally using the same ERM identifier, and then later realizing that the materials are not identical.

Indeed, there are plenty of solutions to handle the equivalence of identifiers. First, identifiers can be merged, which is common in many life sciences databases. While updating outdated identifiers requires maintenance, this maintenance is common practice. Second, the ELIXIR Recommended Interoperability Resource BridgeDb [32] project developed “scientific lenses” within the OpenPHACTS grant [34], a concept that makes the exact equivalence machine readable [33]. For example, it can describe that the connectivity of two small molecules is identical but the structures differ in stereochemistry, or that one molecule is the deprotonated form of the other. This approach is easily applied to nanomaterial identifiers. We can formalize the idea that two nanomaterials are the same material by the same manufacturer but from different synthesis batches. This formalization would be a direct mapping, and when combined with the idea of a parent identifier (see "Registering identifiers" section) would summarize that ontological relation.

A fourth issue is that the information that is coupled to the identifiers is not always publicly available. For some of the above use cases, that information is currently only available within the projects and there is currently no clear solution. Projects have to decide when and how to make this information available. Agreements in the EU NanoSafety Cluster on when to make (meta)data available (for example by default after a project ends) would be very useful. Currently, to make project information open, all partners in the project consortium need to give permission. The RiskGONE project went through this process and added more detail to the corresponding ERM identifiers, setting an example of the added value these open project documents provide (h2020-riskgone.github.io/riskgone-materials/). Without this informationone should not use identifiers reserved by other projects and make assumptions as to which nanomaterials they refer to. Therefore, it is important to keep track of the material’s authority, that is, who can positively identify the material. This can e.g. be a paper or a contact person in the project, but it needs to be linked to the identifier, which is done for most registered materials.

As more projects adopt the ERM, and as its utility and limitations are explored by the nanosafety community, it is planned that community consensus on appropriate uses of the ERM will be defined and documented, and solutions to the issues noted above reached collectively.

## Conclusion

This work describes the European Registry of Materials and the corresponding persistent, unique ERM identifier for nanomaterials and how various nanosafety projects have begun to use it. The identifier fills a need by allowing the projects to apply the FAIR principles to research output while it is being generated. The assignment of ERM identifiers already when selecting nanomaterials to test within a project facilitates the consistent identification of materials by multiple partners within the same project. Tools like eNanoMapper Template Wizard and an integrated ERM lookup on data entry alleviate the problem that ERMs are not easy to remember. It is important to note that how the ERM identifier is used is left to the user currently, and the use cases show examples of how the identifier is adopted. The full value will become clear when many more research outputs are publicly shared, as it will allow us to connect results accurately. The active uptake of the identifier in multiple data reuse case studies, and the intention of projects like NanoInformaTIX and HARMLESS to start using them, bodes well for the future of this persistent nanomaterial identifier.

## Data Availability

Data and approach is available from https://github.com/nanocommons/identifiers. The registry is archived on Zenodo [37].

## Abbreviations

CAS: Chemical Abstracts Service
CEINT: Center for the Environmental Implications of Nanomaterials
CHEMINF: Chemical Information Ontology
dct: RDF namespace prefix for http://purl.org/dc/terms/ for Dublin Core Terms
ENMs: Engineered nanomaterials
ERM: The European Registry of Materials
erm: RDF namespace prefix for https://nanocommons.github.io/identifiers/registry#
FAIR: Findability, accessibility, interoperability, and reusability
foaf: RDF namespace prefix for http://xmlns.com/foaf/0.1/ for the Friend of a Friend ontology
InChI: International Chemical Identifier
IUPAC: The International Union of Pure and Applied Chemistry
MIEs: Molecular Initiating Events
NInChI: InChI for nanomaterials
NSC: The EU Nanosafety Cluster
obo: RDF namespace prefix for http://purl.obolibrary.org/obo/ [35]
PDB: Protein Data Bank
rdfs: RDF namespace prefix for http://www.w3.org/2000/01/rdf-schema#
URI: Uniform Resource Identifier
Wd: RDF namespace prefix for https://www.wikidata.org/entity/ [36]
